# RAS status and neoadjuvant chemotherapy impact CD8+ cells and tumor HLA class I expression in liver metastatic colorectal cancer

**DOI:** 10.1186/s40425-018-0438-3

**Published:** 2018-11-19

**Authors:** Fanny Ledys, Quentin Klopfenstein, Caroline Truntzer, Laurent Arnould, Julie Vincent, Leila Bengrine, Romain Remark, Romain Boidot, Sylvain Ladoire, Francois Ghiringhelli, Valentin Derangere

**Affiliations:** 1 Cancer Biology Research Platform, Centre Georges-François Leclerc, Dijon, France; 2 Université de Bourgogne-Franche comté, Faculté des Sciences de Santé, Dijon, France; 3 GIMI Genetic and Immunology Medical Institute, Dijon, France; 4 Department of Pathology, Centre Georges-François Leclerc, Dijon, France; 5 Department of Medical Oncology, Centre Georges-François Leclerc, Dijon, France; 6 INSERM UMR1231, Dijon, France; 7 Innate Pharma, 117 Avenue de Luminy, Marseille, France

**Keywords:** Colorectal cancer, Liver metastases, CD8, HLA

## Abstract

**Background:**

T lymphocytes and HLA expression on tumor cell both influence prognostic of localized colorectal cancer, but their role following chemotherapy in patients with liver metastatic colorectal cancer (mCRC) was not addressed.

**Methods:**

One hundred fourteen patients treated in curative intend of liver mCRC were included in this retrospective study. Patients were either untreated or treated with neoadjuvant therapy containing an anti-EGFR, bevacizumab or oxaliplatin. Immune densities were quantified in the tumor core and in invasive margin of metastases, using Qupath software or a pathologist’s quantification. CD8, NKp46, Foxp3, CD163, HLA, PD-L1 were analyzed and were correlated with progression free survival (PFS) and overall survival (OS) using multivariable Cox proportional hazards models.

**Results:**

In the whole cohort only a high CD8+ cells infiltrate, a high HLA-I expression and wild-type RAS/RAF status were associated with a better overall survival in both univariate and multivariate model. Moreover, CD8+ cells immune infiltrate at invasive margin combined to HLA expression in cancer cell could increase patient’s outcome prediction. RAS status but not immune cell infiltrate was associated with HLA expression on tumor cells. In comparison to untreated patients, neoadjuvant chemotherapy induced CD8+ cells recruitment and increased PD-L1 staining in immune infiltrates only for WT RAS patients. In this context, anti-EGFR and oxaliplatin based chemotherapy are the most powerful to induce CD8+ cells mobilization within the metastatic site.

**Conclusions:**

While CD8 infiltrate and HLA expression appear to be prognostic for mCRC, CD8 and PD-L1 infiltrate are enhanced by neoadjuvant chemotherapy in mCRC under RAS status dependence.

**Electronic supplementary material:**

The online version of this article (10.1186/s40425-018-0438-3) contains supplementary material, which is available to authorized users.

## Introduction

Colorectal cancer (CRC) is one of the top three most-frequent cancers with 446.000 new cases and 214.000 deaths in Europe each year [1]. About half of the patients diagnosed with CRC will eventually develop liver metastases and only 10–20% of these patients will be resectable resulting in five-year survival rates of up to 60% depending on the tumor characteristics, extent of the disease and resection margin [2]. Considerable progress has been made in the management of metastatic colorectal cancer (mCRC). Resection of CRC metastases, using surgical techniques or local ablation using stereotaxic radiotherapy or radiofrequence could induce complete remission and cure patients. In addition, perioperative chemotherapy using FOLFOX, a combination of 5-fluorouracil, folinic acid and oxaliplatin, was shown to improve progression free survival and became a standard of care for patients with liver metastasis which could be treated with local therapy [3]. However despite optimal therapy many patients develop recurrence. So biomarker to better define patient’s prognosis and to better classify patients are needed.

For a long time, it is known that the immune system plays a crucial role in CRC survival [4]. Tumor infiltrating lymphocytes (TILs) are associated with a better prognosis [5]. In localized tumor, the presence of cytotoxic and memory T cells at the tumor’s invasive margin and tumor’s center could be used to predict survival [6]. Immune infiltrate could be determined using immunoscore [7] and such biomarker is currently validated in large prospective cohort of localized colon cancer [8]. In a cohort of patients with liver metastasis of CRC, the Galon’s group also observed that immunoscore is associated with a poor prognosis, thus suggesting that immune infiltrate in metastasis could also be a surrogate biomarker of clinical outcome [9]. Previous work by our group underlined in preclinical models that chemotherapy could favor CD8 recruitment and activation thus enhancing efficacy of checkpoint inhibitor in preclinical model of MSS (microsatellite stability) colon tumors [10]. However such data are still poorly addressed in human.

To exert their cytotoxic effect CD8 T cells required that, cancer cells present tumor peptide via Human Leucocyte Antigen (HLA) class I molecule [11]. A high expression of HLA class I is associated with a better tumor outcome in localized colorectal cancer [12]. However, such question was not addressed in the setting of mCRC.

In this study, we questioned the prognostic role of immune infiltrate and HLA class I expression in a cohort of 114 French patients suffering from CRC with liver metastasis. We also studied the impact of different neoadjuvant chemotherapeutic regimen on immune infiltrate and tumor microenvironment in order to improve patient’s outcome classification using both clinical and immune parameters.

## Materials and methods

### Statistical analysis

Data analysis was performed using R statistical software (http://www.R-project.org/) and presented with Prism 7 (GraphPad, San Diego, CA, USA). Overall survival was used for survival analysis. Survivors were censored after 80 months. Univariate Cox proportional-hazards models were built to compute hazard ratio with a 95% CI and *p*-values were adjusted using False Discovery Rate (FDR) [13]. Survival curves were estimated using the Kaplan–Meier method and compared using log-rank tests. Multivariate Cox model was also built and FDR applied. The predictive power of these models was compared using Area Under Curve (AUC) parameter. AUC cut-off was set at 40 months to predict model efficacy and was computed using survival ROC R package [14]. Association between qualitative and quantitative variables was tested using Mann-Whitney test. All boxplots were drawn with a median, quartiles and Tukey’s whiskers. Correlation tests and matrix have been also built with Prism. We used the following p-value signification: *: *p < 0.05*; **: *p < 0.01*; ***: *p < 0.001*; ****: *p < 0.0001*; *ns*: not significant.

### Immunohistochemistry procedures and histological analysis

Slides have been stained with a Ventana BenchMark apparatus using antibodies against CD8, HLA-I, PD-L1, CD163, NKp46 and FoxP3. All clones and dilutions used are reported in Additional file 1: Table S4. Each staining was evaluated and quantified respecting whole slide representativeness and complex slides were reviewed by two independent experimented pathologists.

Quantification of CD8 positive cells was performed with QuPath software [15] according to its localization: invasive margin and tumor core. Total CD8+ cells were the sum of these two variables. A mean of 8 different areas of invasive margin representing 3.5 mm^2^ in total for each slide was analyzed and a mean of 14 different areas of tumor core representing 5.6 mm^2^ for each slide was analyzed. The mean of total tissue analyzed for each patient was as a consequence in line with Halama recommendations [16]. To ensure the quality of our analyses and their representative character [8], we also performed a whole liver metastasis analysis for 20 patients increasing the total mean surface analyzed to 46.3 mm^2^.

HLA-I staining has been evaluated by two pathologists independently and separated in three groups: HLA low, HLA intermediate and HLA high. Quantification of HLA-I staining with QuPath software has also been performed to determine an H-score using the following formula: H-score = [1*(% cells 1+) + 2*(% cells 2+) + 3*(% cells 3+)]. The result of this calculation was defined to be the HLA-score. HLA-1 analysis was restrained to tumor cells. A mean of 9 different areas of tumor cells representing 1 mm^2^ for each slide was analyzed for HLA-1 staining.

CD163, NKp46 and Foxp3 were assessed in a semi-quantitative manner by pathologists. PD-L1 was analyzed using classical recommendations based on scores determined by POPLAR [17]. We next clustered in 2 groups: PD-L1 low (grade 0 and 1) and PD-L1 high (grade 2 and 3).

Response rate to chemotherapy is evaluated using *Tumor Regression Grade* (TRG) as previously defined by Rubbia-Brandt et al (2007). Briefly, TRG1 corresponded to an absence of tumor cells replaced by abundant fibrosis; TRG2 corresponded to rare scattered residual tumor cells and abundant fibrosis; TRG3 corresponded to a large amount of residual tumor cells with predominant fibrosis; TRG4 corresponded to tumor cells predominating over fibrosis; and TRG5 corresponded to almost exclusively tumor cells without fibrosis. The mean percentages of *necrosis* and *fibrosis* were quantified as previously reported [18–20].

### Immunofluorescence assay

After dewaxing and rehydration, antigen retrieval in pH 6 citrate buffer (Dako) was performed during 50 min at 95 °C. Saturation was done using 5% normal goat serum during 30 min. Slides were then labelled with primary antibodies anti-CD8 and anti-PD-L1 diluted in PBS/BSA 1% during one hour. After washing in PBS, slides were incubated with secondary antibodies Alexa555-anti-mouse and Alexa647-anti-rabbit (both from Jackson Immunoresearch) diluted at 1/500 in PBS/BSA 1% during 30 min. After washing in PBS-Tween 0.04% three times during 5 min, saturation in PBS/goat serum 5% was performed once again. Slides were then incubated overnight with Alexa488-anti-Ki67 monoclonal antibody and Alexa594-anti-cytokeratin monoclonal antibody. After washing in PBS-Tween 0.04%, spectral DAPI (Perkin Elmer) was applied on slides during 5 min and washed in distilled water. Finally, slides were mounted with Diamond Prolong anti-fade mounting medium (ThermoFischer) and examined using Mantra spectral station (Perkin Elmer) and analysed with inForm software (Perkin Elmer). All clones and dilutions of antibodies used are reported in Additional file 1: Table S4.

## Results

### Patients’ clinical and immune characteristics

**Fig. 1:**
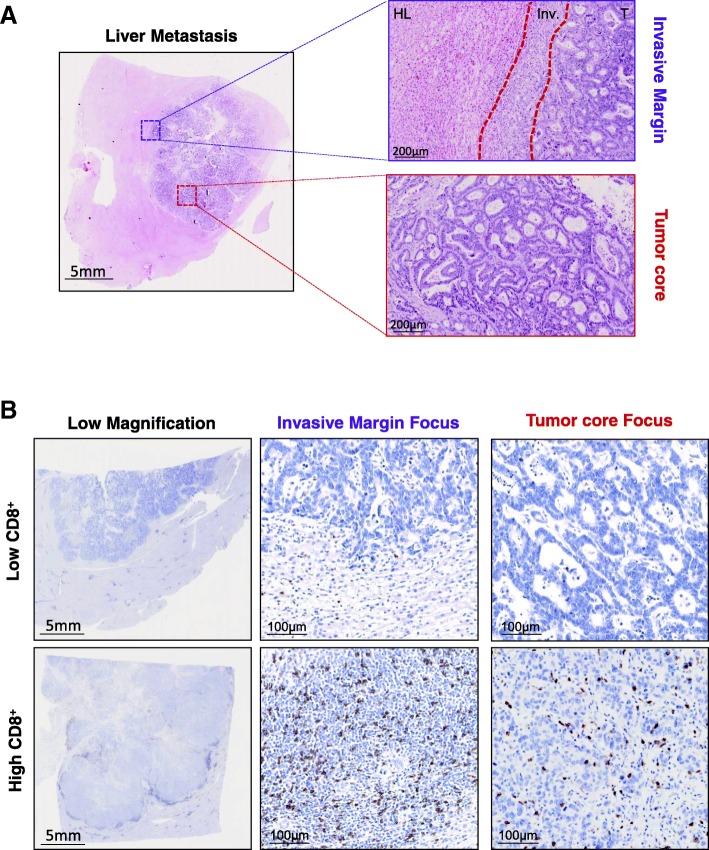
Histological quantification strategy. (**a**) Picture of HES staining of a liver metastasis and inset are focus on invasive margin and tumor core. Scale bar on the left indicates 5 mm and on the right 200 μm. (**b**) Representative pictures of a low IHC CD8 staining; low magnification in the upper left, focus on the invasive margin in the upper middle and tumor core in the upper right. Representative pictures of a high IHC CD8 staining; low magnification in the lower left, focus on the invasive margin in the lower middle and tumor core in the lower right. Scale bar on the left indicates 5 mm and on the right 100 μm (HL: healthy liver, Inv: invasive margin, T: tumor core)

We retrospectively analyzed 114 patients suffering from a colorectal cancer treated in curative intention with surgery of liver metastases. 114 patients have overall survival (OS) available. Median follow-up was 2.9 years. The median age was 63 (29–83). The RAS and BRAF mutational status was determined for all patients and we found 35 patients with RAS mutation, 3 patients with BRAF mutation. Twenty-five patients have right side tumor and 89 patients have left side tumor. Thirty-six were untreated with neoadjuvant chemotherapy. Amongst them, 30 received FOLFOX as adjuvant therapy. Seventy-eight patients were treated with neoadjuvant chemotherapy. Forty seven received FOLFOX as neoadjuvant chemotherapy, 11 patients received FOLFOX plus anti-EGFR (Cetuximab) and 18 were treated with FOLFOX plus bevacizumab. Two patients received FOLFOX plus cetuximab followed by FOLFOX plus bevacizumab due to anti-EGFR side effects (Table 1). For every patient the surgery was performed between 4 and 6 weeks after the last injection of neoadjuvant chemotherapy with a median delay of 36 days. Slides with liver metastasis sections were stained for CD8, HLA-I Foxp3, PD-L1, NKp46 and CD163. For immune parameters, both tumor core and invasive margin were analyzed with the strategy displayed on Fig. 1a. CD8 (Fig. 1b) and HLA class 1were quantified using automated assessment with Qupath software. A complementary whole liver metastasis analysis for CD8^+^ cells was performed for 20 patients and showed very strong correlation between this systematic strategy and our representative strategy for both the invasive margin and the tumor core (Additional file 2: Figure S1A). Generally, invasive margin was more infiltrated than tumor center for each immune population. CD8+ and CD163+ macrophages were the most common immune cells in comparison to NKp46+ natural killer cells (NK) and Foxp3+ regulatory T cells. In our cohort, PD-L1 was mainly expressed by immune cells while PD-L1 expression was observed on tumor cells in only 4 tumors. At the invasive margin NK cells were positively correlated with regulatory T cells density and negatively correlated with CD163+ cells. Within the tumor core, CD8+ cells are positively correlated with PD-L1 and CD163+ cells. CD8+ cells variables (i.e within the tumor core, at the invasive margin and total) were correlated each other (Additional file 2: Figure S1B, C and D). As a result, we only included CD8 at the invasive margin, which was the most significant variable, in the multivariate model.

**Table 1:** Patients’ characteristics summary

Characteristics		Patients (*n* = 114)	
Age		63	(29,83)
Sex	Male	81	71.1
	Female	33	28.9
KRAS mutation	Mutated	35	30.7
	Wild type	79	69.3
BRAF mutation	Mutated	3	2.6
	Wild type	111	97.4
Tumor side	Left	89	78.1
	Right	25	21.9
Chemotherapy	Neoadjuvant	78	68.4
	adjuvant	30	26.3
	None	6	5.2
Type of Chemotherapy	FOLFOX	47	60
	FOLFOX anti VEGF	18	23
	FOLFOX anti EGFR	11	14
	Other	2	3
	Other	45	57.7

### Impact of RAS mutational status on immune environment

**Fig. 2:**
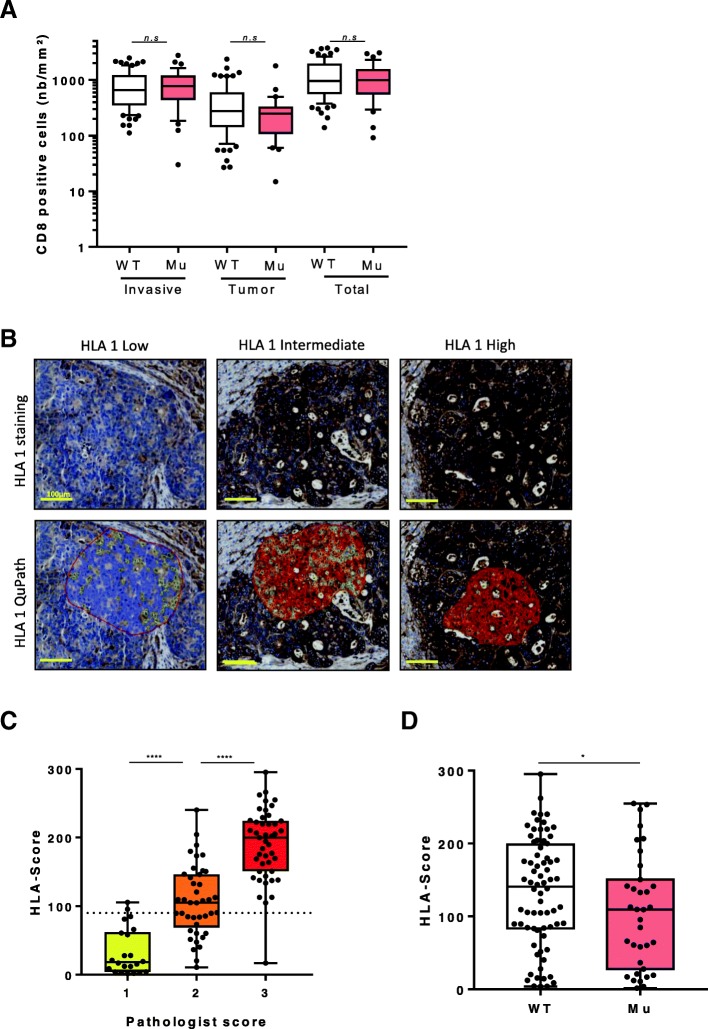
Impact of Ras-Raf mutationnal status on tumor environment. (**a**) Boxplot diagrams presenting the number of CD8 positive cells per mm^2^ according to localization (invasive margin, tumor core and total) in WT patients or with RAS-RAF mutation. (**b**) Representative pictures of HLA1 IHC staining: HLA1 low in the upper left, HLA1 intermediate in the upper middle and HLA1 in the upper right. Representative pictures of HLA1 quantification using QuPath software; HLA1 1+ (yellow) in the lower left, HLA1 2+ (orange) the lower middle and HLA1 3+ (red) in the lower right. Scale bar indicates 100 μm. (**c**) Boxplot diagrams presenting association between HLA-score determined by QuPath and the pathologist’s score. (**d**) Boxplot diagrams presenting the HLA-score according to mutational status (WT: wild type, Mu: mutated, *n.s*: not significant, *: *p* < 0.05, **: *p* < 0.01, ****: *p* < 0.0001)

RAS mutation is a well-known factor associated with resistance to anti-EGFR and with a poorer prognosis. In our cohort RAS mutation is as expected related to a poorer clinical outcome in term of OS (HR = 1.67 IC95% [1.03–2.73] *p* = 0.04 for OS) (Additional file 2: Figure S2A and Table 2) but not related to PFS (HR = 1.25 IC95% [0.79–1.97] *p* = 0.34 for PFS) (Additional file 1: Table S1). We addressed the impact of RAS mutational status on immune infiltrate. We observed that CD8+ cells infiltrate in the tumor margin or in the tumor core is not affected by RAS mutational status (Fig. 2a). Moreover we did not find any association between RAS status on PD-L1 expression, CD163, NKp46 or FOXP3 infiltrate, suggesting that RAS mutational status do not impact in situ immune response (Additional file 2: Figure S2B & C and Additional file 1: Table S2 & S3). We also performed quantification of HLA-I expression (Fig. 2b) using IHC procedures and semi-automated computer assisted quantification (see Methods). We named that continuous variable “HLA score” which was perfectly correlated with pathologists’ bins quantification (Fig. 2c). HLA class I expression on tumor cells is homogenous on a same metastasis. Only 4 tumors have no HLA expression on cancer cells. In contradiction with other immune parameters, constitutive RAS activation is associated with reduced HLA-I expression on tumor cells (Fig. 2d). HLA stayed independent with all other immune markers evaluated (Additional file 2: Figure S2D). Together, such data demonstrate that RAS status does not directly impact on immune infiltrate in metastasis but is associated with a decrease in HLA-I expression on tumor cells which could subsequently impair antigen presentation ability of cancer cells.

**Table 2:** Overall Survival statistical summary

Variable		Total	Univariate Cox models				Multivariate Cox models															
							Clinical model				Clinical and CD8 model				Clinical and HLA model				Clinical and HLA/CD8 model			
			Hratio	95% IC	*p*	adj padj padj p	Hratio	95% IC	p	adj padj padj p	Hratio	95% IC	p	adj padj padj p	Hratio	95% IC	p	adj padj padj p	Hratio	95% IC	p	adj padj padj p
Age (%)	median (range)	63 (29–83)	1.015	[0.993;1.038]	*0.18*	0.42	1.018	[0.995;1.041]	0.13	0.36	1.012	[0.990;1.035]	0.3	0.57	1.016	[0.993;1.040]	0.17	0.42	1.015	[0.993;1.039]	0.18	0.42
continuous	mean(sd)	61.73 (11.47)																				
Sex(%)	Female	33 (28.95)	1				1				1				1				1			
	Male	81 (71.05)	0.762	[0.469;1.238]	*0.27*	0.55	0.77	[0.464;1.282]	0.32	0.57	0.771	[0.460;1.292]	0.32	0.57	0.759	[0.447;1.289]	0.31	0.57	0.686	[0.397;1.185]	0.18	0.42
Chemo(%)	No	36 (31.58)	1				1				1				1				1			
	Yes	78 (68.42)	0.812	[0.502;1.314]	*0.4*	0.6	0.73	[0.432;1.231]	0.24	0.53	0.928	[0.534;1.612]	0.79	0.92	0.744	[0.438;1.262]	0.27	0.55	0.793	[0.464;1.355]	0.4	0.6
KRAS	No	79 (69.30)	1				–				–				–				–			
	Yes	35 (30.70)	1.979	[1.211;3.233]	*0.006*	0.05																
BRAF+KRAS	No	76 (66.67)	1				1				1				1				1			
	Yes	38 (33.33)	1.737	[1.07;2.82]	*0.03*	0.13	2.06	[1.223;3.487]	0.007	0.05	2.171	[1.281;3.682]	0.004	0.05	1.882	[1.104;3.230]	0.02	0.1	1.716	[0.992;2.968]	0.05	0.2
Tumor side	Right	25 (21.93)	1				1				1				1		1		1			
	Left	89 (78.07)	0.92	[0.528;1.604]	*0.77*	0.91	1.304	[0.721;2.359]	0.38	0.6	1.151	[0.639;2.074]	0.64	0.79	1.334	[0.735;2.421]	0.34	0.58	1.208	[0.671;2.175]	0.53	0.7
CD8 invasive margin continuous			0.66	[0.500;0.866]	*0.003*	0.05					0.644	[0.477;0.869]	0.004	0.05								
CD8 tumor core continuous			0.906	[0.679;1.209]	*0.5*	0.67																
CD8 total continuous			0.705	[0.534;0.926]	*0.01*	0.06																
HLA score		41 (37.27)	1												1							
		69 (62.73)	0.593	[0.366;0.959]	*0.03*	0.13									0.631	[0.379;1.050]	0.08	0.25				
HLA/CD8 combined	Low/Low	24 (21.82)	1																1			
	Others	86 (78.18)	0.391	[0.230;0.663]	*0.0005*	0.02													0.415	[0.234;0.737]	0.003	0.05
PDL1 TC SP142	High	18	1																			
	Low	5 (4.39)	0.6	[0.147;2.443]	*0.48*	0.66																
PDL1 invasive margin SP142	High	34 (29.82)	1																			
	Low	80 (70.18)	0.791	[0.463;1.348]	*0.39*	0.6																
CD163 invasive margin	1	27 (23.68)	1																			
	2	42 (36.84)	0.719	[0.408;1.266]	*0.25*	0.54																
	3	32 (28.07)	0.807	[0.443;1.467]	*0.48*	0.66																
FoxP3 invasive margin	0	45 (39.47)	1																			
	1	20 (17.54)	1.182	[0.654;2.135]	*0.58*	0.73																
NKp46	Low	50 (49.50)	1																			
	High	45 (50.50)	1.024	[0.640;1.636]	*0.93*	0.98																
AUC		–	–				0.63				0.72				0.67				0.72

### Combination of CD8+ cells and HLA score could be used to predict survival

**Fig. 3:**
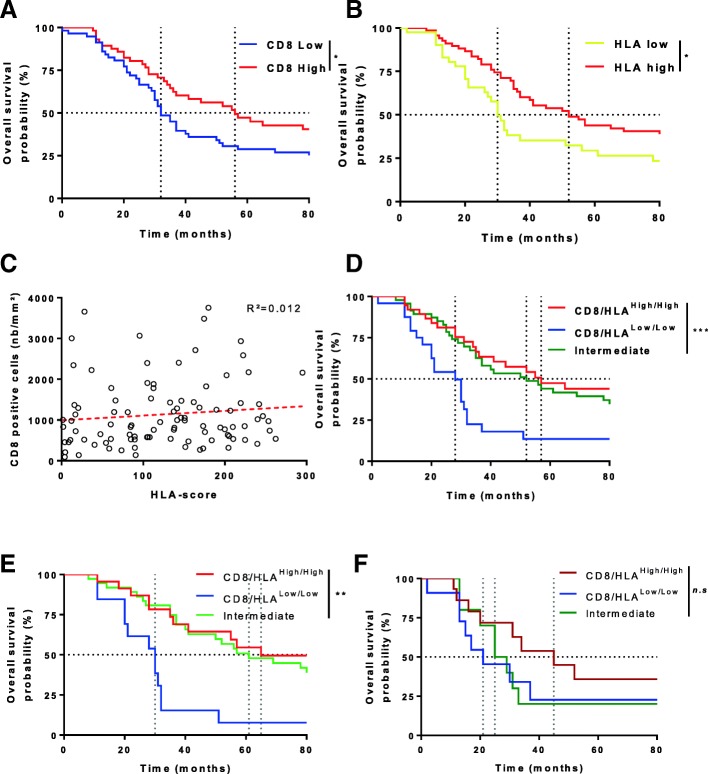
HLA score and CD8 positive cells combination as prognostic factor. (**a**) Kaplan-Meier survival analysis based on the number (low (*n* = 57) or high (n = 57) split at the median) of CD8 positive cells per mm^2^. (**b**) Kaplan-Meier survival analysis based on the HLA-score (low (*n* = 41) or high (*n* = 69)). (**c**) Correlation between HLA- score and the number of CD8 positive cells per mm^2^. (**d**) Kaplan-Meier survival analysis based on the composite variable using CD8+ cells and HLA-score (low/low in blue (*n* = 24), intermediate in green (*n* = 47), high/high in red (*n* = 38)) (**e**) Kaplan-Meier of overall survival analysis based on the composite variable CD8/HLA for WT patients (low/low in blue (*n* = 13), intermediate in green (*n* = 37), high/high in red (*n* = 23). (**f**) Kaplan-Meier of overall survival analysis based on the composite variable CD8/HLA for RAS-RAF mutated patients (low/low in dark blue (*n* = 11), intermediate in dark green (*n* = 10), high/high in dark red (*n* = 15). (*: p < 0.05, **: p < 0.01, ***: *p* < 0.001)

Using CD8 as continuous variable with a Cox univariate model we tested the prognostic role of CD8+ cells at the invasive margin, within the tumor core or total in terms of OS (Table 2) and PFS (Additional file 1: Table S1). Only a high count of CD8 total or at the invasive margin was associated with a better outcome in OS and remained significant in PFS. Using this variable separated by the median we observed using Kaplan-Meier curves that CD8 infiltrate at the invasive margin is associated with a better OS (Fig. 3a). HLA score variable was split in two groups to separate patients with low HLA staining (pathologist’s subgroup 1). HLA score was positively associated with OS (Table 2, Fig. 3b). Other immune variables were classified as dichotomous variables and were not linked with OS nor PFS (Table 2, Additional file 1: Table S1). As there was no correlation between CD8+ cells and HLA score (Fig. 3c) we suspected that both variables could bring complementary information. We subsequently tested the combination of CD8+ cells and HLA score on patients’ survival. We split patients in 3 groups, i.e. high^CD8/HLA^, low^CD8/HLA^, or intermediate with only one highly expressed biomarker. The same cutoffs as above were selected. The best survival rates were observed in the double high and the intermediate groups while the poorer outcome was observed in the double low group (Fig. 3d). Subgroup analysis was also performed according to RAS status. It confirmed the better prognosis of high^CD8/HLA^ in WT patients (Fig. 3e) and a clear-cut but non-significant trend for RAS-mutated patients (Fig. 3f). We also found that high^CD8/HLA^ WT and mutated patients shared a similar outcome (Additional file 2: Figure S3A). The same observation was done in low^CD8/HLA^ WT and mutated patients (Additional file 2: Figure S3B). Interestingly, we observed a strong difference of outcome in the intermediate groups for WT patients compared to RAS mutated group (Additional file 2: Figure S3C). Upon Cox multivariate model, only RAS status and CD8 cell density at the invasive margin remained independently associated with OS (Table 2). We next tested the capacity of different multivariate model to discriminate prognosis. We compared a pure clinical model, a model that included clinical variable and CD8+ cells at invasive margin, a model combining clinical and HLA variables and finally, a model that include the CD8/HLA variables and clinical variable. After correcting *p*-values using False Discovery Rate (FDR) method, AUC underlined that the model including CD8/HLA and clinical variables was the best model to discriminate prognostic in term of OS (Table 2) [13]. All in all our data suggest that the combination of CD8+ cells and HLA score is a powerful prognostic tool for people suffering from liver metastasis from CRC.

### Role of neoadjuvant chemotherapy status on immune infiltrate

**Fig. 4:**
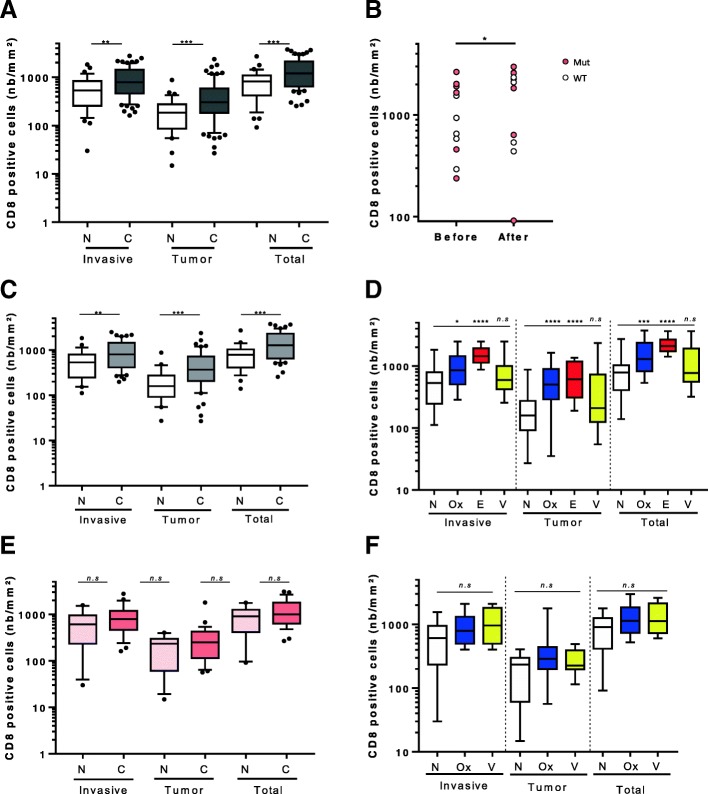
Impact of neoadjuvant chemotherapy on immune infiltrates. (**a**) Boxplot diagrams presenting number of CD8 positive cells per mm^2^ in all patients separated according to their localization (invasive margin, tumor core and total). (**b**) Graphics showing the number of CD8+ cells per mm^2^ before and after neoadjuvant chemotherapy. “Before” is the diagnostic liver biopsy while “after” is the metastasis surgery following neoadjuvant procedure. (**c**)(**d**) Boxplot diagrams presenting number of CD8 positive cells per mm^2^ in patients with wild type RAS status and the neoadjuvant treatment separated according to localization (invasive margin, tumor core and total) and the treatment. (**e**)(**f**) Boxplot diagrams presenting number of CD8 positive cells per mm^2^ in patients with RAS mutated status and the neoadjuvant treatment separated according to localization (invasive margin, tumor core and total) and the treatment (N: no neoadjuvant chemotherapy, C: neoadjuvant chemotherapy, Ox: oxaliplatin, E: anti-EGFR, V: anti-VEGF, WT: wild type, Mu: mutated, *n.s*: not significant, *: p < 0.05, **: p < 0.01, ***: p < 0.001, ****: p < 0.0001)

We then addressed the impact of neoadjuvant chemotherapy on immune infiltrate in liver metastasis of CRC. While PD-L1 expression was slightly but significantly increased within the tumor core, we noted that neoadjuvant (NA) chemotherapy did not impact NKp46, CD163 and Foxp3 infiltrate. NA procedures did not influence HLA-I expression nor PD-L1 at the invasive margin (Additional file 2: Figure S4A and B). In contrast, a higher CD8+ cells count was observed for patients treated with neoadjuvant chemotherapy both at the invasion margin and in the tumor compared to untreated patients (Fig. 4a). For 11 mCRC patients, a liver biopsy before neoadjuvant administration as well as a metastasis surgery after neoadjuvant procedure was available. We also observed for these patients an increase of CD8+ cells infiltrate in tumor site after neoadjuvant therapy (Fig. 4b) thus confirming that neoadjuvant chemotherapy increased CD8+ cells in liver mCRC in a paired-samples model. Response to neoadjuvant chemotherapy was evaluated using TRG classification. We did not observe any difference in tumor HLA-score or CD8 accumulation neither in tumor core nor at the invasive margin between responders and none responders (Additional file 2: Figure S4C). Because RAS mutational status is a major prognostic and predictive factor which drives therapeutic care, we separated on one hand RAS wild type patients and RAS mutated patients on the other hand. In wild-type RAS patients, we observed that neoadjuvant chemotherapy enhanced CD8+ cell infiltrate both at the invasion margin and in the tumor center (Fig. 4c). When we focused on the different type of neoadjuvant chemotherapy regimen within the RAS wild type group, we observed that oxaliplatin-based neoadjuvant chemotherapy and anti-EGFR-based neoadjuvant chemotherapy were highly efficient to induce CD8+ cells accumulation. On the contrary, neoadjuvant chemotherapy containing anti-VEGF weakly induced CD8+ cells recruitment (Fig. 4d). Even if not significant, a slight trend was observed for a better OS for patients treated with anti-EGFR (Additional file 2: Figure S4D) while their HLA-score remained analogous compared to other NA regimen (Additional file 2: Figure S4E). Surprisingly in RAS mutated tumors, chemotherapy was unable to induce CD8+ cells recruitment in both invasion margin and in tumor center (Fig. 4e). Even if a slight trend was observed, notably for oxaliplatin-based NA chemotherapy, no significant difference with control patients occurred (Fig. 4f).

Together these observations underline that oxaliplatin and anti-EGFR-based chemotherapy induce CD8-T cells recruitment only in Wild Type RAS tumors.

### Tumor stroma becomes immunosuppressive after NA CD8+ mobilizing chemotherapy

**Fig. 5:**
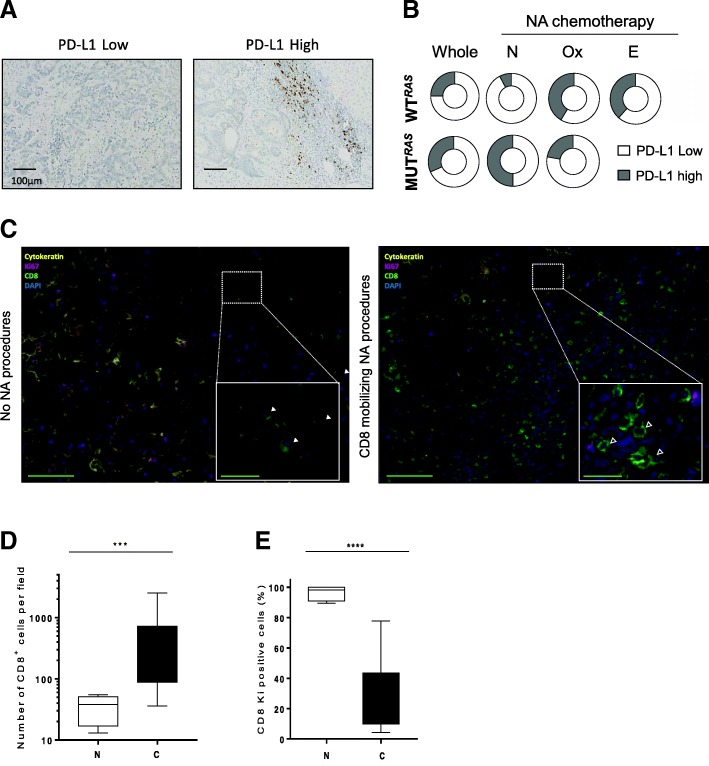
CD8+ cells’ recruitment modifies tumor stroma making mCRC elligible for immune therapeutic intervention. (**a**) Representative pictures of PD-L1 [SP142] IHC staining: left panel with a low PD-L1 staining PD-L1 and right panel with a high PD-L1 staining. Scale bar indicates 250 μm. (**b**) Donut holes representing WT or patients with RAS-RAF mutated status according to different neoadjuvant chemotherapy. (**c**) Representative pseudocolored (Dapi in blue, CD8 in lime green, Ki67 in magenta and CK in yellow) pictures of immunofluorescence staining of liver metastasis (scale bar = 150 μm). Inset is a focus showing CD8/Ki67 double positive cells (green and magenta pointed with white arrowhead) on the left panel and Ki67 negative CD8+ cells (empty arrowhead) on the right panel (scale bar = 30 μm). (**d**) Quantification of CD8 T cells according to their NA procedures. (**e**) Quantification of CD8/Ki67 double positive cells in patients according to their neoadjuvant procedure (NA: neoadjuvant, N: no neoadjuvant chemotherapy, C: CD8 mobilizing chemotherapy, ***: p < 0.001, ****: p < 0.0001)

As CD8+ cells were increased in RAS WT patients under oxaliplatin-based or EGFR-based neoadjuvant chemotherapy, we hypothesized that CD8+ cells could enhanced PD-L1 expression via interferon secretion. All surgery pieces were stained with anti-PD-L1 SP142 clone, which detect PD-L1 expression in both cancer cells and immune cells. The staining was, in almost cases, restrained to tumor associated myeloid cells found in the stroma surrounding or within tumor core (Fig. 5a). In RAS wild type patients who underwent NA we observed a higher expression of PD-L1 in comparison to untreated patients. In contrast, PD-L1 expression was not impacted by chemotherapy in RAS mutated patients (Fig. 5b). To go further, we performed multispectral analysis to detect proliferating CD8-T cells using Ki67 labelling and PD-L1 expression on the same slide in some RAS WT patients untreated with NA or who underwent NA chemotherapy procedure with oxaliplatin or anti-EGFR-based chemotherapy (Fig. 5c). We observed an increase in CD8+ cells infiltration and PD-L1 expression on myeloid cells following NA chemotherapy (Fig. 5d and Additional file 2: Figure S5) but a strong a decrease in CD8/Ki67 double positive cells (Fig. 5e). Together, our data suggest that WT patients who underwent NA chemotherapy, CD8+ cells were firstly recruited but a negative feedback loop occurred with a decrease in their proliferation.

**Fig. 6:**
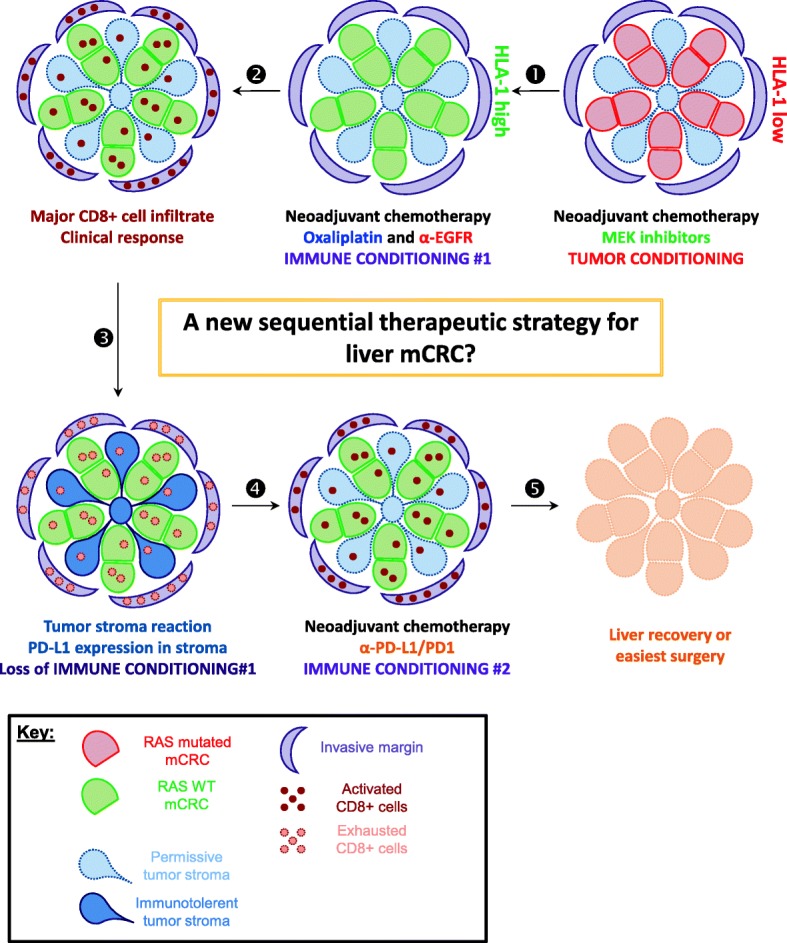
Graphical abstract: A new sequential therapeutic strategy for liver mCRC? **(1)** Patients with KRAS mutated are HLA class 1 low. Neoadjuvant chemotherapy like MEK inhibitors could increase HLA-I expression in the tumor core. **(2)** Neoadjuvant chemotherapy like oxaliplatin and anti-EGFR can induce an immune conditioning with an increase in CD8+ cells infiltrate in tumor core, invasive margin and tumor stroma, giving a clinical response. **(3)** Increase in CD8+ cells within the tumor subsequently induces PD-L1 expression and a loss of immune conditioning. **(4)** As a result, ICP anti-PD1/PD-L1 could be considered to restore an efficient cytotoxic immune response leading to a liver recovery or easiest surgery **(5)**

## Discussion

Our study underlines the prognostic role of HLA expression and CD8 infiltrate in mCRC. We also observed that RAS status impact HLA expression in such patients. Moreover our study shows that oxaliplatin and anti-EGFR-based chemotherapy are effective therapy to promote CD8 recruitment in WT RAS tumor. However, this recruitment of cytotoxic lymphocytes is associated with an induction of PD-L1 expression by myeloid cells subsequently inhibiting CD8+ cell proliferation.

Treatment of CRC with metastasis restricted to liver is well codified and includes neoadjuvant chemotherapy followed by surgical resection of residual disease [3]. Neoadjuvant chemotherapy could lower the tumor burden and so facilitate surgery procedures and improve patients’ outcome. It is now well established that immune infiltrates play a major impact on patients’ prognosis in primary site of colorectal tumor [8, 21, 22]. However, few things are known on immune infiltrates in liver metastasis of CRC and their evolution under neoadjuvant chemotherapy procedures. Baseline CD8 infiltrate in liver metastasis was reported to be associated with better prognosis by Gallon’s group [9]. In addition, good response to treatment was significantly associated with high-immune densities in metastasis. Similarly in EORTC 40983 study, a high CD3+ lymphocytes infiltrate appear to be prognostic and associated with pathological response to FOLFOX chemotherapy [2]. However, the role of the type of chemotherapy and of classical clinical prognostic factor like RAS mutational status on immune response was still not addressed. We took advantage of our 114 mCRC with clinical database paired with liver surgery to investigate these questions.

Our retrospective evaluation of patients suffering from mCRC is firstly dedicated to the evaluation of RAS implication on liver mCRC survival and immune infiltrates. We confirmed that a constitutive activation of RAS pathway play a critical role in patients’ progression free survival and overall survival. CD8+ T cells are widely known to be correlated with a better prognostic notably in colon cancer. Our results confirmed these positive effects of CD8+ cells on OS in the setting of mCRC, notably at the invasive margin. Despite their same effect on patients’ prognostic, CD8 immune infiltrate and RAS status were not correlated in our study. Moreover no other immune cells infiltrate is correlated with RAS status. In contrast, RAS mutated tumors displayed a lower expression of HLA-I. Because HLA-I is required for antigen recognition by CD8-T cells we could suspect that RAS mutated tumors will appear less sensitive to CD8 infiltrate. Accordingly, a recent paper from Liu [23] reported that CD8+/CD3+ cells infiltrates are only prognostic on RAS WT patients. In contrast, in our cohort CD8+ cells are prognostic in both WT and mutated RAS patients, thus suggesting that despite a low HLA-I expression RAS mutated tumor are still controlled by adaptive immune response. However, even if few patients were available, the subgroups analysis showed that only patients with the combination of intermediate markers (i.e only HLA-I high or CD8 high) have a significant worst OS in RAS mutated patients compared to their counterpart WT. Other works showed that constitutive RAS activation impacted HLA-I expression in vitro and in preclinical models [24]. In such models Mek inhibitor restore HLA-I expression on murine colon cancer cells. One can think that applying such a pressure to express HLA-I with therapeutic could create a tumor conditioning promoting immune response (Fig. 6, part 1).

In the second part of our study, we focused on the impact of neoadjuvant chemotherapy regimen on immune infiltrates in liver mCRC. We observed that in RAS WT patients under oxaliplatin-based or anti-EGFR-based neoadjuvant chemotherapy had a major impact on CD8+ cells recruitment in liver. A recent report from Inoue et al. also showed that CD8+ and CD3+ cells were recruited under anti-EGFR neoadjuvant procedures on a small cohort of Japanese liver mCRC patients [25]. Anti-EGFR efficacy is dependent of NK cells in this paper. In contrast, we did not find any increase in NK cells following anti-EGFR therapy but an important CD8-T cells recruitment. In addition, NK cells are not associated with outcome in our study. We also observed that oxaliplatin-based neoadjuvant therapy induced CD8+ cells mobilization in WT RAS patients. As Inoue did not observe any impact of oxaliplatin-based neoadjuvant therapy, we could subsequently hypothesize that RAS mutated patients were over-represented in this cohort treated without anti-EGFR. Our observation on CD8+ cells recruitment was confirmed on paired biopsies and liver surgeries from patients treated with anti-EGFR or oxaliplatin-based chemotherapy. While such analysis could be biased by immune infiltrate heterogeneity a recent technological paper showed that CD8+ T-cell evaluation by immunohistochemistry for colorectal cancer by tumor biopsy fragments is a valuable representation of immune infiltrate within the whole tumor [26]. Previous reports from Galon and Pagès used a similar strategy to test immune infiltrates in liver metastasis of colorectal cancer and rectum before any treatment using a biopsy and after therapy using pathological sample with similar result with immune cells accumulation after therapy [9, 27]. As a result, we believe that WT patients should be treated with neoadjuvant therapy containing oxaliplatin or anti-EGFR to create a cytotoxic immunologic microenvironment (Fig. 6, part 2).

However, one consequence of CD8-T cells recruitment in tumor environment is a potential induction of adaptive tolerance. As activated CD8-T cells secrete IFN gamma (IFNg), cells expressing its receptor can activate Jak2/Stat1/4 pathway. Stat1/4 can upregulate immune checkpoint (ICP) expression such as PD-L1 [28] (graphical abstract part 3). Consequently, we observed an increase in PD-L1 expression on myeloid cells in patients with a high CD8+ cells count in their tumor environment. In an interesting manner, we showed that WT RAS patients who received mobilizing NA procedures had less CD8/Ki67 double positive cells compared to untreated patients. Our data underline that CD8+ cells exhaustion is probably related to increase PD-L1 expression. Work is still required with immunomonitoring to precise chronology between activation and exhaustion of CD8+ cells in mCRC context. This immune adaptive tolerance may partially explain the poor prognosis of mCRC even for patients that received immunogenic chemotherapies and clearly give a rational to add checkpoint inhibitors with neoadjuvant therapy at least for WT RAS patients (Fig. 6, part 4).

To conclude, our study shows that liver mCRC can benefit from immune recruitment following neoadjuvant chemotherapy. Limitations of our study came from its monocentric and retrospective design. The large time span inclusion of patients can induce some bias in OS analysis. Our results indicated that RAS WT patients are those who get the more important immune efficacy when treated with oxaliplatin-based or anti-EGFR-based therapy with an increase in CD8+ count, a high HLA-I expression and a subsequent PD-L1 induction. Such data could support that RAS mutated tumor may be poor candidates for immunotherapy in case of a low HLA expression and suggest the rational of to associate oxaliplatin-based chemotherapy or anti-EGFR in association with ICP inhibitors for RAS WT patients. Subgroups analysis could have been more interesting and informative if more patients could have been analyzed in the FOLFOX-EGFR arm. Another limitation of our study is the method of quantification of immune parameters such as CD8^+^ cells in representative areas. Although our results were in total concordance with whole slide analysis on 20 patients, whole slide analyses should be performed on any surgery piece to have a better reflect on liver metastasis heterogeneity. As a consequence our exploratory study clearly requires a prospective multicentric validation before considering any further trials combining immunotherapy and chemotherapy in this metastatic context of CRC.

## Additional files

Additional file 1: **Table S1.** Progression Free Survival statistical summary. **Table S2.** Overall Survival statistical summary for WT patients. **Table S3.** Overall Survival statistical summary for RAS-RAF mutated patients. **Table S4.** List of antibodies used. (XLSX 20 kb)

Additional file 2: **Figure S1.** Immune parameters correlation. **Figure S2.** Impact of Ras-Raf mutationnal status tumor environment. **Figure S3.** HLA score and CD8 positive cells combination as prognostic factor. **Figure S4.** Impact of neoadjuvant chemotherapy on immune infiltrates. **Figure S5.** CD8+ cells’ recruitment modifies tumor stroma making mCRC elligible for immune therapeutic intervention. (ZIP 430 kb)

## Abbreviations

AUC: Area under curve
CRC: Colorectal cancer
EGFR: Epidermal grow factor receptor
FOLFOX: 5-fluorouracil, folinic acid, oxaliplatin
HLA: Human leucocyte antigen
INFg: Interferon gamma
mCRC: Metastatic colorectal cancer
MSS: Microsatellite stability
NA: Neadjuvant
NK: Natural killer
OS: Overall survival
PFS: Progression free survival
TILs: Tumor infiltrating lymphocytes
WT: Wild-type
