# Supplementary material for: RAS status and neoadjuvant chemotherapy impact CD8+ cells and tumor HLA class I expression in liver metastatic colorectal cancer
Source: J Immunother Cancer. 2018 Nov 19;6:123. doi: 10.1186/s40425-018-0438-3 (PMC6245855; doi:10.1186/s40425-018-0438-3)

Supplemental Figure 1 : Immune parameters correlation

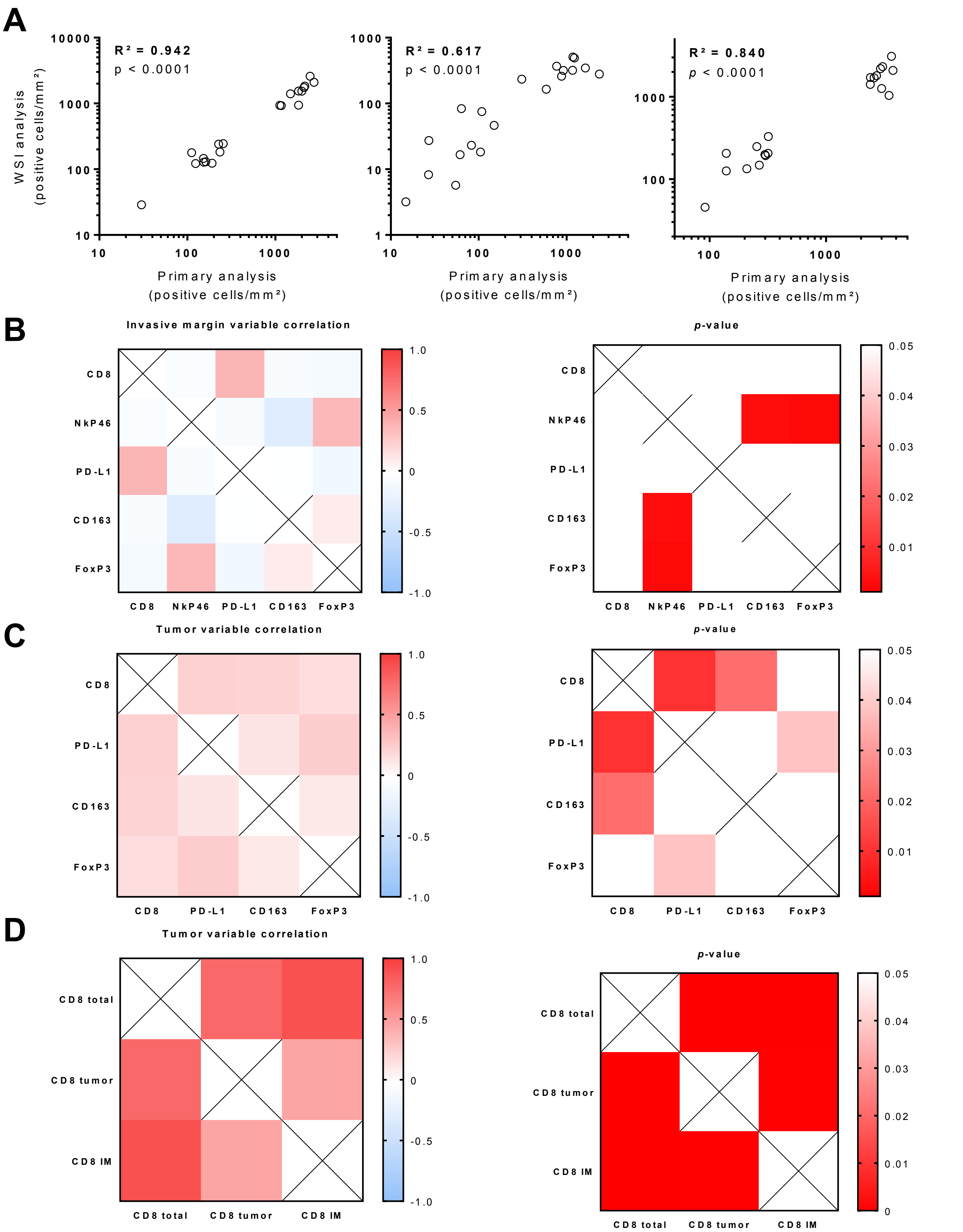

Supplemental Figure 2 : Impact of Ras-Raf mutationnal status tumor environment

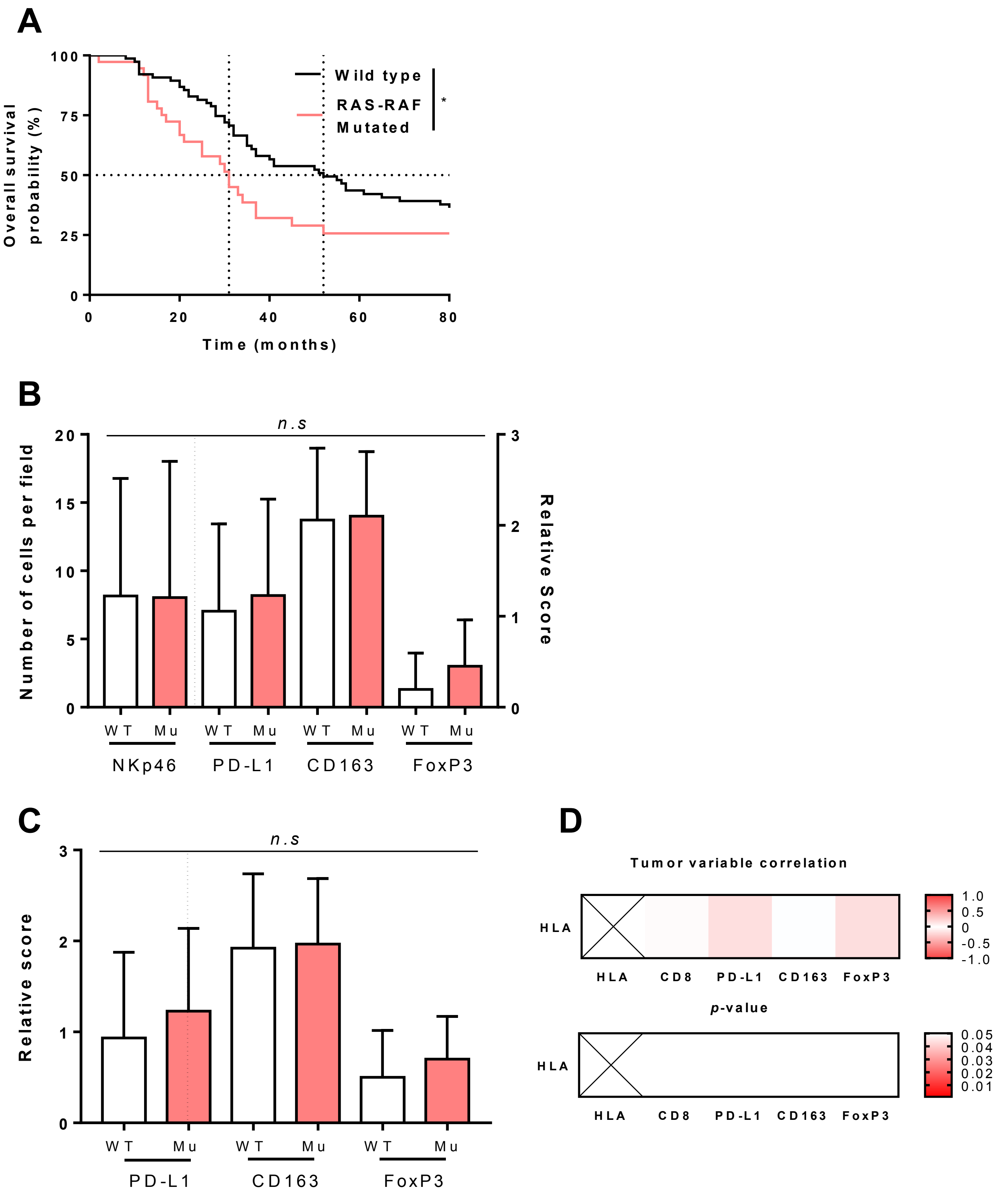

Supplemental Figure 3 : HLA score and CD8 positive cells combination as prognostic factor

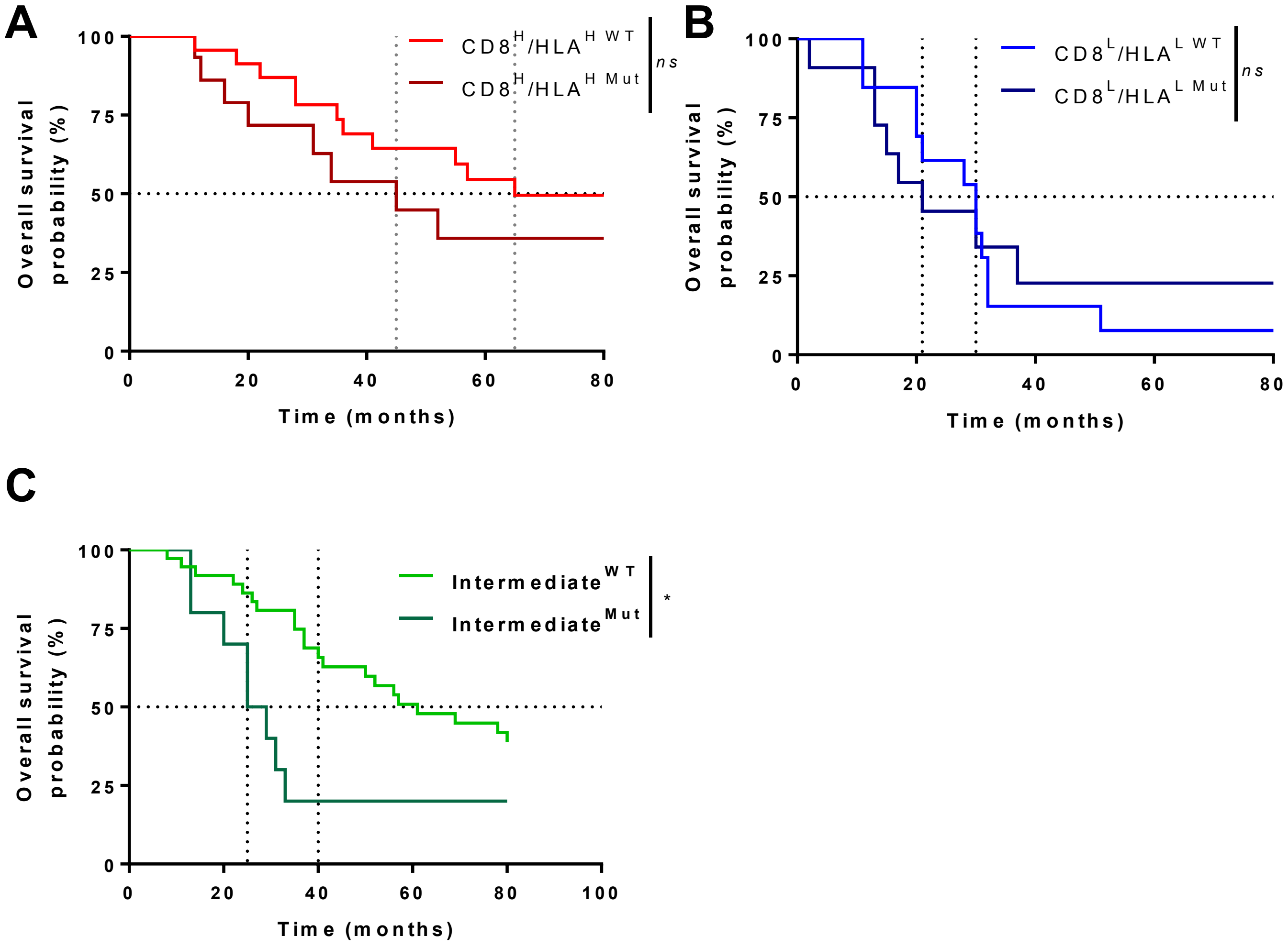

Supplemental Figure 4 : Impact of neoadjuvant chemotherapy on immune infiltrates

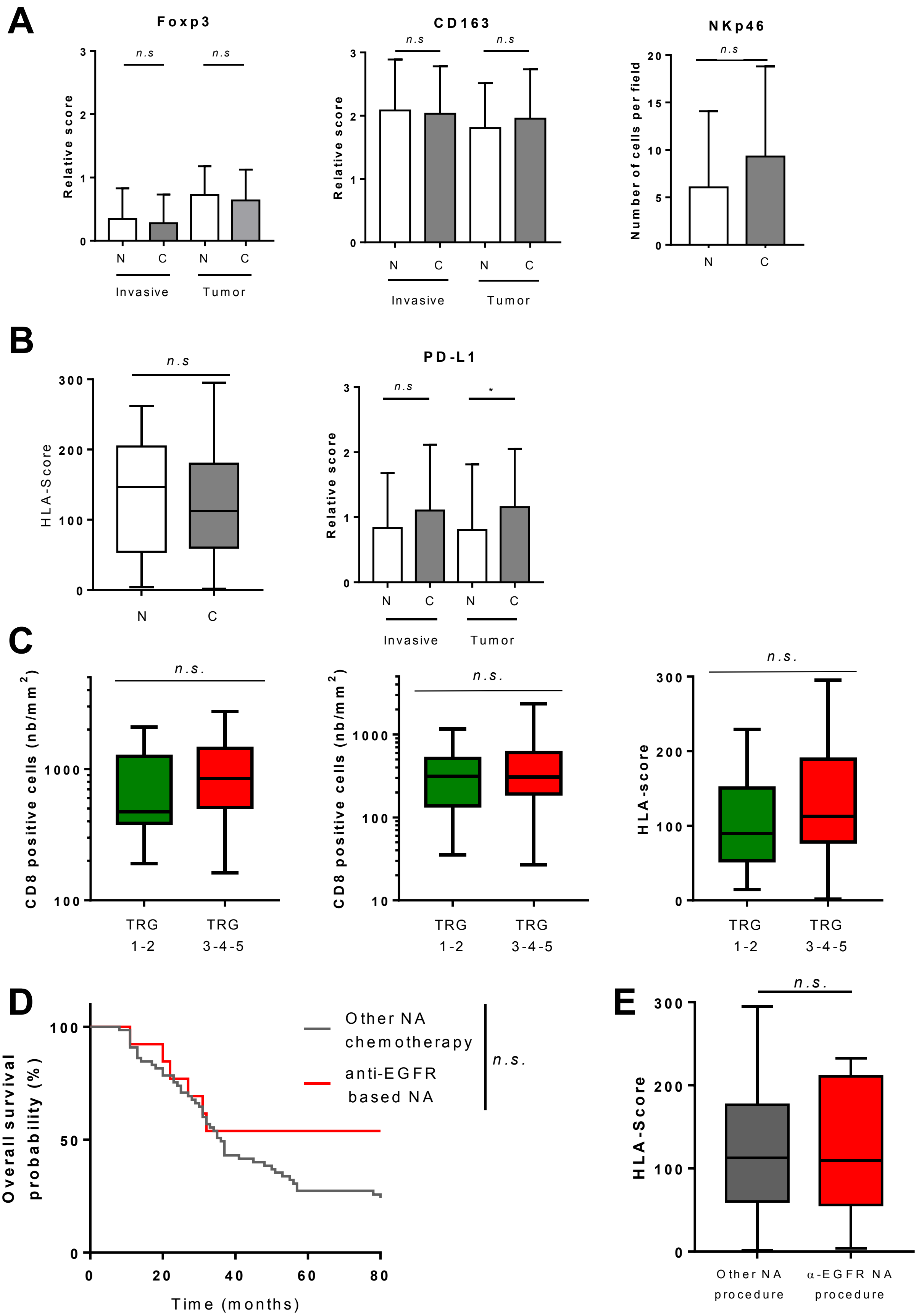

Supplemental Figure 5 : CD8+ cells' recruitment modifies tumor stroma making mCRC elligible for immune therapeutic intervention

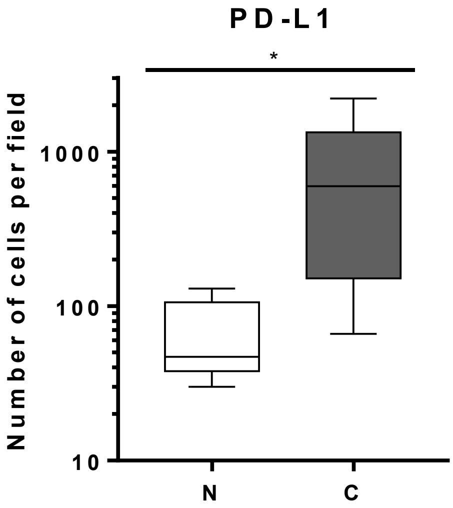

Supplement: Supplementary file 2 — Figure S1. Immune parameters correlation. Figure S2. Impact of Ras-Raf mutationnal status tumor environment. Figure S3. HLA score and CD8 positive cells combination as prognostic factor. Figure S4. Impact of neoadjuvant chemotherapy on immune infiltrates. Figure S5. CD8+ cells’ recruitment modifies tumor stroma making mCRC elligible for immune therapeutic intervention. (ZIP 430 kb) [file 40425_2018_438_MOESM2_ESM.zip › Ledys_et_al_SDR2.pdf]
