# Supplementary material for: RAS status and neoadjuvant chemotherapy impact CD8+ cells and tumor HLA class I expression in liver metastatic colorectal cancer
Source: J Immunother Cancer. 2018 Nov 19;6:123. doi: 10.1186/s40425-018-0438-3 (PMC6245855; doi:10.1186/s40425-018-0438-3)
Supplement: Supplementary file 2 — Figure S1. Immune parameters correlation. Figure S2. Impact of Ras-Raf mutationnal status tumor environment. Figure S3. HLA score and CD8 positive cells combination as prognostic factor. Figure S4. Impact of neoadjuvant chemotherapy on immune infiltrates. Figure S5. CD8+ cells’ recruitment modifies tumor stroma making mCRC elligible for immune therapeutic intervention. (ZIP 430 kb) [file 40425_2018_438_MOESM2_ESM.zip › Ledys_revised_figure_captions_R2.docx]

**Supplemental Figure 1: Immune parameters correlation**. **(A) Correlation between CD8^+^ cells quantification using whole slide analyses or primary quantification at the invasive margin (left panel), within the tumor core (middle panel) or total (right panel). R² correlation score is displayed with p-value for each location. (WSI: Whole Slide Analysis). (B)** Correlation matrix between immune populations: CD8, NkP46, PD-L1, CD163 and FoxP3 at the invasive margin. Positive correlation is in red and negative correlation is in blue. The correlation matrix next to it represents its p-values. **(C)** Correlation matrix between immune populations: CD8, NkP46, PD-L1, CD163 and FoxP3 in the tumor core. Positive correlation is in red and negative correlation is in blue. The correlation matrix next to it represents its p-values. **(D)** Correlation matrix between CD8 variables. Positive correlation is in red and negative correlation is in blue. The correlation matrix next to it represents its p-values.

**Supplemental Figure 2: Impact of Ras-Raf mutationnal status tumor environment.** **(A)** Kaplan-Meier survival analysis based on the RAS-RAF mutational status (WT (n= 76), Mu (n=38)). **(B)** Diagrams representing number of cells per field or relative score of immune populations NKp46, PD-L1, CD163, FoxP3 according to mutational status localized at the invasive margin. **(C)** Diagrams representing relative score of immune populations PD-L, CD163, FoxP3 according to mutational status localized within the tumor core. **(D)** Correlation matrix between immune parameters with HLA-score. The correlation matrix under it represents its p-values (WT: wild type, Mu: mutated, *n.s*: not significant).

**Supplemental Figure 3: HLA score and CD8 positive cells combination as prognostic factor. (A)** Kaplan-Meier of overall survival analysis based on the composite variable CD8^high^/HLA^high^ WT (light red, n=23) and RAS-RAF mutated (dark red, n=15). **(B)** Kaplan-Meier of overall survival analysis based on the composite variable CD8^low^/HLA^low^ WT (light blue, n=13) and RAS-RAF mutated (dark blue, n=11). **(C)** Kaplan-Meier of overall survival analysis based on the composite variable intermediate CD8/HLA WT (light green, n=37) and RAS-RAF mutated (dark green, n=10) patients (WT: wild type, Mut: mutated*, n.s*: not significant, *: p<0.05).

**Supplemental Figure 4: Impact of neoadjuvant chemotherapy on immune infiltrates.** **(A)** Diagrams representing relative score of immune populations FoxP3, CD163 and NKp46 according neoadjuvant chemotherapy or not and localization (invasive margin, tumor core or total). **(B)** Diagrams representing relative score of HLA-score and PD-L1 according neoadjuvant chemotherapy or not and localization (invasive margin, tumor core or total). **(C)** Boxplot diagrams representing number of CD8 positive cells per field according to TRG grade at the invasive margin (left panel) and in tumor core (center panel). HLA-score according to TRG grade (right panel) (n=18 for low TRG and n=60 for high TRG. **(D)** Kaplan-Meier survival analysis based on neo-adjuvant chemotherapy (anti-EGFR (n=13, red line) or other NA (n=65, dark grey line)) (E) Boxplot diagrams presenting HLA-score according to NA procedures (other NA in dark grey (n=62) and anti-EGFR in red (n=13) (*n.s*: not significant, *: p<0.05).

**Supplemental Figure 5: CD8+ cells’ recruitment modifies tumor stroma making mCRC elligible for immune therapeutic intervention.** Boxplot diagrams representing number of PD-L1 positive cells per field according to neoadjuvant chemotherapy analyzed by immunofluorescence (N: no neoadjuvant chemotherapy, C: CD8 mobilizing chemotherapy, *: p<0.05).
